# Sedimentation of a Charged Soft Sphere within a Charged Spherical Cavity

**DOI:** 10.3390/molecules29133087

**Published:** 2024-06-28

**Authors:** Yong-Jie Lin, Huan J. Keh

**Affiliations:** Department of Chemical Engineering, National Taiwan University, Taipei 10617, Taiwan; r11524103@ntu.edu.tw

**Keywords:** sedimentation velocity, charged soft particle, charged cavity, electrokinetics, boundary effect

## Abstract

The sedimentation of a soft particle composed of an uncharged hard sphere core and a charged porous surface layer inside a concentric charged spherical cavity full of a symmetric electrolyte solution is analyzed in a quasi-steady state. By using a regular perturbation method with small fixed charge densities of the soft sphere and cavity wall, a set of linearized electrokinetic equations relevant to the fluid velocity field, electrical potential profile, and ionic electrochemical potential energy distributions are solved. A closed-form formula for the sedimentation velocity of the soft sphere is obtained as a function of the ratios of core-to-particle radii, particle-to-cavity radii, particle radius-to-Debye screening length, and particle radius-to-porous layer permeation length. The existence of the surface charge on the cavity wall increases the settling velocity of the charged soft sphere, principally because of the electroosmotic enhancement of fluid recirculation within the cavity induced by the sedimentation potential gradient. When the porous layer space charge and cavity wall surface charge have the same sign, the particle velocity is generally enhanced by the presence of the cavity. When these fixed charges have opposite signs, the particle velocity will be enhanced/reduced by the presence of the cavity if the wall surface charge density is sufficiently large/small relative to the porous layer space charge density in magnitude. The effect of the wall surface charge on the sedimentation of the soft sphere increases with decreases in the ratios of core-to-particle radii, particle-to-cavity radii, and particle radius-to-porous layer permeation length but is not a monotonic function of the ratio of particle radius-to-Debye length.

## 1. Introduction

The sedimentation of charged particles in ionic fluids under gravity is a common phenomenon in various fields of colloidal science, as well as biomedical, mechanical, chemical, civil, and environmental engineering [1,2]. This phenomenon is more complex than the migration of uncharged particles because the ambient fluid flow distorts the electrical double layer surrounding each charged particle and induces a sedimentation potential gradient [3,4]. This induced electric field alters the velocity distribution of the ionic fluid via electrostatic interaction and diminishes the settling velocity of the charged particle by an electrophoretic effect.

Using a regular perturbation method with the particle zeta potential as a small perturbation parameter, Booth [5] first obtained analytical formulas for the sedimentation velocity and potential in suspensions of hard (impermeable to the ionic fluid) spheres with arbitrary double layer thickness as power expansions in their low zeta potential. Numerical results relaxing the assumption of low zeta potential in this analysis were calculated by Stigter [6]. Ohshima et al. [7] obtained analytical formulas and numerical results of the sedimentation velocity and potential in suspensions of hard spheres for a wide range of zeta potential and double-layer thickness. On the other hand, the regular perturbation analyses have been extended to obtain the sedimentation velocity and potential in concentrated suspensions of interacting hard spheres [8,9,10,11,12], porous (permeable to the ionic fluid) spheres [13,14], and soft (hard core covered with porous surface layer) spheres [15,16]. The equations that govern the ionic concentration, electric potential, and fluid flow fields around each charged particle migrating in an electrolyte solution are linearized, assuming that the system is only slightly distorted from the equilibrium. These linearized equations are solved with the fixed charge densities of the particles as the small perturbation parameters. A unit cell model that allows for the overlap of the electric double layers of adjacent particles is used to account for the particle-interaction effects. The sedimentation velocity and potential are not necessarily monotonic functions of the volume fraction of the particles. The particle concentration effects are significant, even in dilute suspensions.

In various applications of sedimentation, colloid particles are rarely unbounded but often settle close to solid boundaries [17,18]. In the past, the boundary effect on sedimentation of uncharged particles were studied extensively [19,20,21,22,23], but not much information of this effect on charged particles was conveyed. Pujar and Zydney [24] calculated the settling velocity of a charged spherical particle in a concentric uncharged spherical cavity using perturbation expansions of small zeta potentials and Peclet number. This study has been extended to numerical calculations in the case of arbitrary zeta potentials [25]. More recently, the settling of a charged hard [26] or porous [27] spherical particle within a concentric charged spherical cavity with arbitrary electric double layers has been analytically investigated for the case of small fixed charge densities of the particle and cavity wall. 

In this paper, the sedimentation of a charged soft spherical particle inside a concentric charged spherical cavity with arbitrary double-layer thickness is analytically studied. The fluid velocity, electric potential, and ionic electrochemical potential energy distributions satisfying the linearized electrokinetic equations are determined as a power series of the small fixed charge densities of the soft sphere and cavity wall. A closed-form formula for the settling velocity of the soft sphere is obtained as a function of relevant parameters. 

## 2. Electrokinetic Equations

**Figure 1:**
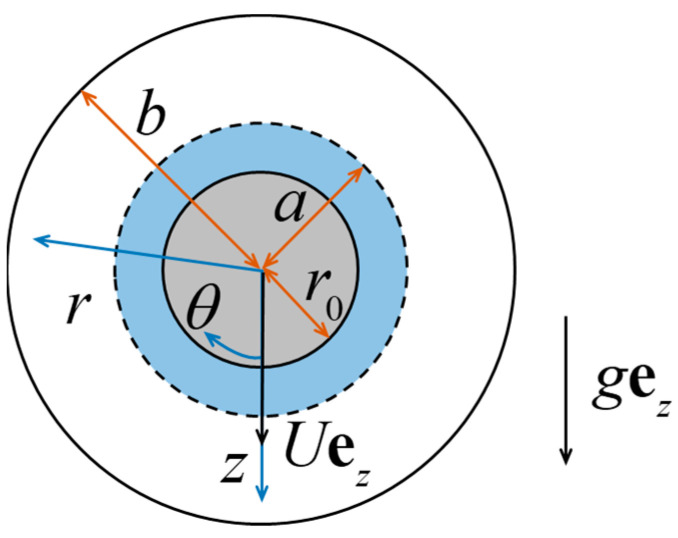
Geometric sketch for the sedimentation of a soft sphere inside a concentric spherical cavity.

As illustrated in Figure 1, we consider the quasi-steady sedimentation of a soft sphere of radius a, consisting of an uncharged hard sphere core of radius $r_{0}$ and a charged porous surface layer of thickness $a-r_{0}$, inside a concentric charged spherical cavity of radius b full of the fluid solution of a symmetric electrolyte. The porous surface layer is permeable to the electrolyte solution and has fixed charges distributed at a uniform space density. The gravitational acceleration field $ge_{z}$ ($e_{z}$ is the unit vector in the z direction) is acting on the system, and the sedimentation velocity $Ue_{z}$ of the soft sphere will be determined. The origin of the spherical coordinates (r,θ,ϕ) is attached to the particle/cavity center (at z=0), and the system is independent of ϕ (symmetric about the z axis).

### 2.1. Differential Equations

The system is assumed to deviate slightly from the equilibrium. Therefore, the pressure profile p(r,θ), electrical potential field ψ(r,θ), and ionic concentration distributions $n_{+}(r,\theta )$ and $n_{-}(r,\theta )$ can be decomposed into

(1a) $$p=p^{\left(\mathrm{eq}\right)}+\delta p,$$

(1b) $$\psi =\psi^{\left(\mathrm{eq}\right)}+\delta \psi ,$$

(1c) $$n_{\pm }=n_{\pm }^{\left(\mathrm{eq}\right)}+\delta n_{\pm },$$

respectively, where $p^{\left(\mathrm{eq}\right)}(r,\theta )$, $\psi^{\left(\mathrm{eq}\right)}(r)$, and $n_{\pm }^{\left(\mathrm{eq}\right)}(r)$ are the equilibrium pressure, electrical potential, and ionic concentrations, respectively, [$n_{\pm }^{\left(\mathrm{eq}\right)}(r)$ and $\psi^{\left(\mathrm{eq}\right)}(r)$ are related by the Boltzmann equation], δp(r,θ), δψ(r,θ), and $\delta n_{\pm }(r,\theta )$ are the small perturbed quantities to the relevant equilibrium fields, and the subscripts + and − denote the cation and anion, respectively.

The small perturbations δp, δψ, $\delta n_{\pm }$, and fluid velocity field u(r,θ) satisfy the continuity equation of the incompressible fluid (∇⋅u=0) following linearized electrokinetic equations [13]:

(2) $$\eta [\nabla^{2}-h(r)\lambda^{2}]u=\nabla \delta p-\varepsilon [\nabla^{2}\psi^{\left(\mathrm{eq}\right)}\nabla \delta \psi +\nabla^{2}\delta \psi \nabla \psi^{\left(\mathrm{eq}\right)}]-h(r)Q\nabla \delta \psi ,$$

(3) $$\nabla^{2}\delta \psi =\frac{Zen^{\infty }}{\varepsilon kT}[\exp(\frac{Ze\psi^{\left(\mathrm{eq}\right)}}{kT})(\delta \mu_{-}+Ze\delta \psi )-\exp(-\frac{Ze\psi^{\left(\mathrm{eq}\right)}}{kT})(\delta \mu_{+}-Ze\delta \psi )],$$

(4) $$\nabla^{2}\delta \mu_{\pm }=\pm \frac{Ze}{kT}\{\nabla \psi^{\left(\mathrm{eq}\right)}\cdot \nabla \delta \mu_{\pm }-\frac{kT}{D_{\pm }}\nabla \psi^{\left(\mathrm{eq}\right)}\cdot u\},$$

resulting from the modified Stokes/Brinkman equation, Poisson’s equation, and continuity equations of the ionic species, respectively. In Equations (2)–(4), $\delta \mu_{\pm }(r,\theta )$ are the perturbed quantities of the ionic electrochemical potential energies defined in terms of δψ and $\delta n_{\pm }$,

(5) $$\delta \mu_{\pm }=\pm Ze\delta \psi +\frac{kT}{n_{\pm }^{\left(\mathrm{eq}\right)}}\delta n_{\pm },$$

η and ε are the viscosity and dielectric permittivity of the fluid, respectively, $n^{\infty }$ and Z are the bulk concentration and valence, respectively, of the symmetric electrolyte, $D_{\pm }$ is the diffusivity of the ionic species, 1/λ is the square root of the permeability of the fluid in the porous surface layer of the soft sphere, h(r) equals unity if $r_{0}\leq r\leq a$ and zero, otherwise k is Boltzmann’s constant, T is the absolute temperature, and e is the charge of a proton. For porous particles made of plastic foam slab [28] and steel wool [29], experimental data for 1/λ can be 400 microns, while in the surface layers of grafted polymer microcapsules [30], rat lymphocytes [31], and human erythrocytes [32] in electrolyte solutions, 1/λ was found as low as 3 nm.

### 2.2. Boundary Conditions

The boundary conditions of the perturbations u, δψ, and $\delta \mu_{\pm }$ at the interface between the hard sphere core and the porous surface layer, as well as at the particle surface, are [15,33,34]

(6) $$r=r_{0}:\;u=0,\;e_{r}\cdot \nabla \delta \psi =0,\;e_{r}\cdot \nabla \delta \mu_{\pm }=0,$$

(7) $$r=a:\;u,\;e_{r}\cdot \tau ,\;\delta \psi ,\;\nabla \delta \psi ,\;\delta \mu_{\pm },\;\mathrm{and}\;\nabla \delta \mu_{\pm }\;\mathrm{are}\;\mathrm{continuous},$$

where τ is the viscous stress and $e_{r}$ is the unit vector in the r direction. Equation (7) represents the continuity requirements of the fluid velocity and stress, electrical potential and field, as well as ionic concentrations and fluxes at the interface. Various boundary conditions describing fluid flow at interfaces between porous media and surrounding fluids are discussed in detail in the literature [35,36] related to Darcy’s law and Brinkman’s equation, and Equation (7) is physically true and mathematically consistent with Equation (2).

The boundary conditions of the small perturbations at the cavity wall are [26,27]

(8) $$r=b:\;u=-Ue_{z},\;e_{r}\cdot \nabla \delta \psi =0,\;e_{r}\cdot \nabla \delta \mu_{\pm }=0.$$

Equations (6) and (8) take a reference frame moving with the particle and show that the Gauss condition holds at the surfaces of the hard sphere core and cavity wall, while no ions can penetrate into these surfaces.

## 3. Solution of Electrokinetic Equations

### 3.1. Equilibrium Electric Potential

For the electrolyte solution around a soft spherical particle whose porous surface layer has a uniform space charge density Q located at the center of a spherical cavity with a constant surface charge density σ, the equilibrium electric potential profile satisfying proper boundary conditions was obtained as [37]

(9) $$\psi^{\left(\mathrm{eq}\right)}=\psi_{\mathrm{eq}01}(r)\bar{Q}+\psi_{\mathrm{eq}10}(r)\bar{\sigma },$$

where

$$\psi_{\mathrm{eq}01}(r)=\frac{kTe^{-\kappa r}}{2ZeA\kappa r}\{[e^{\kappa a}(\kappa a-1)(\kappa r_{0}+1)-e^{\kappa (2r_{0}-a)}(\kappa a+1)(\kappa r_{0}-1)][e^{2\kappa b}(\kappa b-1)$$

(10a) $$+e^{2\kappa r}(\kappa b+1)]\}\;\mathrm{for}\;a<r<b,$$

$$\psi_{\mathrm{eq}01}(r)=\frac{kTe^{\kappa (2b-a+2r_{0}-r)}}{2ZeA\kappa r}\{\frac{1}{A}+e^{\kappa (a-2b)}(\kappa b+1)(\kappa r_{0}-1)[e^{\kappa a}(\kappa a-1)-2e^{\kappa r}\kappa r]$$

$$-e^{\kappa (r-2r_{0})}(\kappa b-1)(\kappa r_{0}+1)[e^{\kappa r}(\kappa a+1)-2e^{\kappa a}\kappa r]+e^{2\kappa (a-b-r_{0}+r)}(\kappa a-1)(\kappa b+1)$$

(10b) $$\times (\kappa r_{0}+1)+\kappa [b-r_{0}(\kappa b-1)-a(\kappa b-1)(\kappa r_{0}-1)]\}\;\mathrm{for}\;r_{0}<r<a,$$

(11) $$\psi_{\mathrm{eq}10}(r)=\frac{2kT{\left(\kappa b\right)}^{2}e^{\kappa (b+r_{0})}}{ZeA\kappa r}\{\kappa r_{0}\cosh[\kappa (r-r_{0})]+\sinh[\kappa (r-r_{0})]\},$$

(12) $$A=e^{2\kappa b}(\kappa b-1)(\kappa r_{0}+1)-e^{2\kappa r_{0}}(\kappa b+1)(\kappa r_{0}-1),$$

$\bar{Q}=ZeQ/\varepsilon \kappa^{2}kT$ and $\bar{\sigma }=Ze\sigma /\varepsilon \kappa kT$ are the dimensionless fixed charge densities, and $\kappa^{-1}={\left(\varepsilon kT/2Z^{2}e^{2}n_{0}^{\infty }\right)}^{1/2}$ is the Debye screening length. Note that the contributions of the second-order fixed charge densities (${\bar{Q}}^{2}$, $\bar{Q}\bar{\sigma }$, and ${\bar{\sigma }}^{2}$) to $\psi^{\left(\mathrm{eq}\right)}$ in Equation (9) vanish for the case of symmetric electrolytes, and higher-order contributions are not needed for the calculation of the particle sedimentation velocity to the second orders.

Experimental data on porous surface layers of poly(*N*-isopropylacrylamide) hydrogels [38], rat lymphocytes [31], and human erythrocytes [32] in electrolyte solutions show that Q has the magnitude of $10^{7}\;C/m^{3}$. For σ, experimental studies on AgI surfaces in an aqueous solution show that it ranges from 0 to 0.035 $C/m^{2}$ when pAg increases from 5.6 to 11 [39]. For the system of soft particles inside a cavity filled with the aqueous solution of monovalent electrolytes with $\kappa^{-1}=1\;\mathrm{nm}$, $Q=10^{7}\;C/m^{3}$, and $\sigma =0.01\;C/m^{2}$, the dimensionless fixed charge densities $\bar{Q}≅\bar{\sigma }≅0.5$ are obtained.

Substituting Equations (9)–(12) into the Gauss condition at r=b, we obtain the following relation between the zeta potential ζ and the surface charge density σ of the cavity wall confining the soft sphere:

$$\{\kappa r_{0}\cosh[\kappa (b-r_{0})]+\sinh[\kappa (b-r_{0})]\}\kappa^{2}b\sigma$$

$$=\{\kappa (b-r_{0})\cosh[\kappa (b-r_{0})]+(\kappa^{2}br_{0}-1)\sinh[\kappa (b-r_{0})]\}\varepsilon \kappa^{2}\zeta$$

(13) $$-\{\kappa (a-r_{0})\cosh[\kappa (a-r_{0})]+(\kappa^{2}ar_{0}-1)\sinh[\kappa (a-r_{0})]\}Q.$$

Namely, $\psi^{\left(\mathrm{eq}\right)}$ expressed by Equation (9) after substituting Equation (13) is still valid for the case of constant zeta potential at the cavity wall.

### 3.2. Small Perturbations

To solve the small perturbations u, δp, δψ, and $\delta \mu_{\pm }$ in terms of the particle velocity U for small dimensionless charge densities $\bar{Q}$ and $\bar{\sigma }$, these variables are expressed as perturbed expansions in powers of $\bar{Q}$ and $\bar{\sigma }$ up to the second orders, such as

(14) $$U=U_{00}+U_{01}\bar{Q}+U_{10}\bar{\sigma }+U_{02}{\bar{Q}}^{2}+U_{11}\bar{Q}\bar{\sigma }+U_{20}{\bar{\sigma }}^{2},$$

where the coefficients $U_{ij}$ with i and j equal to 0, 1, or 2. They are determined to be independent of $\bar{Q}$ and $\bar{\sigma }$ but are functions of the ratios of core-to-particle radii $r_{0}/a$, particle-to-cavity radii a/b, particle radius-to-Debye length κa, and particle radius-to-porous-layer-permeation length λa. In the expansions of δψ and $\delta \mu_{\pm }$, there is no zeroth-order term of $\bar{Q}$ and $\bar{\sigma }$ because no ionic concentration gradient or electric field is applied.

Substituting the expansions of u, δp, δψ, $\delta \mu_{\pm }$, and U in the form of Equation (14) and Equation (9) for $\psi^{\left(\mathrm{eq}\right)}$ into Equations (2)–(8), we obtain the following solution for the components of u in spherical coordinates δp (to the orders ${\bar{Q}}^{2}$, $\bar{Q}\bar{\sigma }$, and ${\bar{\sigma }}^{2}$), δψ, and $\delta \mu_{\pm }$ (to the orders $\bar{Q}$ and $\bar{\sigma }$):

$$u_{r}=\{F_{00r}(r)[U_{00}+U_{01}\bar{Q}+U_{10}\bar{\sigma }]+[U_{02}F_{00r}(r)+U_{00}F_{02r}(r)]{\bar{Q}}^{2}$$

(15a) $$+[U_{11}F_{00r}(r)+U_{00}F_{11r}(r)]\bar{Q}\bar{\sigma }+[U_{20}F_{00r}(r)+U_{00}F_{20r}(r)]{\bar{\sigma }}^{2}\}\cos\theta ,$$

(15b) $$u_{\theta }=-\frac{\tan\theta }{2r}\frac{d}{dr}(r^{2}u_{r}),$$

$$\delta p=\frac{\eta }{a}\{U_{00}F_{p00}(r)+U_{01}F_{p00}(r)\bar{Q}+U_{10}F_{p00}(r)\bar{\sigma }$$

$$+[U_{02}F_{p00}(r)+U_{00}F_{p02}(r)+\frac{a\varepsilon \kappa^{2}}{\eta }U_{00}\psi_{\mathrm{eq}01}(r)F_{\psi 01}(r)]{\bar{Q}}^{2}$$

$$+[U_{11}F_{p00}(r)+U_{00}F_{p11}(r)+\frac{a\varepsilon \kappa^{2}}{\eta }U_{00}\{\psi_{\mathrm{eq}01}(r)F_{\psi 10}(r)+\psi_{\mathrm{eq}10}(r)F_{\psi 01}(r)\}]\bar{Q}\bar{\sigma }$$

(15c) $$+[U_{20}F_{p00}(r)+U_{00}F_{p20}(r)+\frac{a\varepsilon \kappa^{2}}{\eta }U_{00}\psi_{\mathrm{eq}10}(r)F_{\psi 10}(r)]{\bar{\sigma }}^{2}\}\cos\theta ,$$

(16) $$\delta \psi =U_{00}[F_{\psi 01}(r)\bar{Q}+F_{\psi 10}(r)\bar{\sigma }]\cos\theta ,$$

(17) $$\delta \mu_{\pm }=ZeU_{00}[F_{01\pm }(r)\bar{Q}+F_{10\pm }(r)\bar{\sigma }]\cos\theta .$$

Here, the functions $\psi_{\mathrm{eq}01}(r)$ and $\psi_{\mathrm{eq}10}(r)$ have been provided by Equations (10) and (11), and the functions $F_{ijr}(r)$, $F_{pij}(r)$, $F_{\psi ij}(r)$, and $F_{ij\pm }(r)$ are provided by Equations (A1)–(A4), (A9), and (A10) in Appendix A. Because $F_{\psi 01}(r)$, $F_{\psi 10}(r)$, $F_{01\pm }(r)$, and $F_{10\pm }(r)$ are influenced by the zeroth-order fluid flow field, the leading orders of the relaxation effect on the electrical double layers adjacent to the particle and cavity wall are contained in the solutions for δψ and $\delta \mu_{\pm }$ to the first orders $\bar{Q}$ and $\bar{\sigma }$ (which are sufficient for calculations of the settling velocity to the second orders ${\bar{Q}}^{2}$, $\bar{Q}\bar{\sigma }$, and ${\bar{\sigma }}^{2}$).

### 3.3. Forces on the Particle

The net force exerted on the soft spherical particle undergoing sedimentation includes the gravity, electrical, and hydrodynamic forces. The gravity, which has nothing to do with the fixed and mobile electric charges, is

(18) $$F_{g}=\frac{4}{3}\pi [{r_{0}}^{3}(\rho_{c}-\rho )+(a^{3}-{r_{0}}^{3})(1-\varepsilon_{p})(\rho_{p}-\rho )]ge_{z},$$

where $\rho_{p}$ and $\varepsilon_{p}$ are the mass density and porosity, respectively, of the surface layer of the particle, ρ and $\rho_{c}$ are the mass densities of the fluid and hard core, respectively, and $ge_{z}$ is the gravitational acceleration. 

The electrical force acting on the charged soft particle is [26,37]

(19) $$F_{e}=2\pi a^{2}\varepsilon \int_{0}^{\pi }{\left[\nabla \delta \psi \nabla \psi^{\left(\mathrm{eq}\right)}\right]}_{r=a}\;\cdot e_{r}\sin\theta d\theta .$$

Substitution of Equations (9) and (16) into Equation (19) results in

$$F_{e}=\frac{4}{3}\pi \varepsilon aU_{00}\{[2F_{\psi 01}(a)\frac{d\psi_{\mathrm{eq}01}}{dr}(a)+a\frac{dF_{\psi 01}}{dr}(a)\frac{d\psi_{\mathrm{eq}01}}{dr}(a)]{\bar{Q}}^{2}+[2F_{\psi 01}(a)\frac{d\psi_{\mathrm{eq}10}}{dr}(a)$$

$$+a\frac{dF_{\psi 01}}{dr}(a)\frac{d\psi_{\mathrm{eq}10}}{dr}(a)+2F_{\psi 10}(a)\frac{d\psi_{\mathrm{eq}01}}{dr}(a)+a\frac{dF_{\psi 10}}{dr}(a)\frac{d\psi_{\mathrm{eq}01}}{dr}(a)]\bar{Q}\bar{\sigma }$$

(20) $$+[2F_{\psi 10}(a)\frac{d\psi_{\mathrm{eq}10}}{dr}(a)+a\frac{dF_{\psi 10}}{dr}(a)\frac{d\psi_{\mathrm{eq}10}}{dr}(a)]{\bar{\sigma }}^{2}\}e_{z}.$$

Only the second orders ${\bar{Q}}^{2}$, $\bar{Q}\bar{\sigma }$, and ${\bar{\sigma }}^{2}$ contribute to $F_{e}$, since both δψ (sedimentation-induced electric potential) and $\psi^{\left(\mathrm{eq}\right)}$ are of the first orders $\bar{Q}$ and $\bar{\sigma }$.

The hydrodynamic drag force exerted on the soft particle is provided by

(21) $$F_{h}=2\pi a^{2}\int_{0}^{\pi }{\left\{\eta [\nabla u+\nabla {\left(u\right)}^{T}]\cdot e_{r}-\delta pe_{r}\right\}}_{r=a}\sin\theta d\theta .$$

Substitution of Equation (15) into the previous equation leads to

$$F_{h}=-4\pi \{\eta aC_{006}(U_{00}+U_{01}\bar{Q}+U_{10}\bar{\sigma })$$

$$+[\eta a(C_{026}U_{00}+C_{006}U_{02})+\frac{1}{3}\varepsilon {\left(\kappa a\right)}^{2}U_{00}F_{\psi 01}(a)\psi_{\mathrm{eq}01}(a)]{\bar{Q}}^{2}$$

$$+[\eta a(C_{116}U_{00}+C_{006}U_{11})+\frac{1}{3}\varepsilon {\left(\kappa a\right)}^{2}U_{00}\{F_{\psi 10}(a)\psi_{\mathrm{eq}01}(a)+F_{\psi 01}(a)\psi_{\mathrm{eq}10}(a)\}]\bar{Q}\bar{\sigma }$$

(22) $$+[\eta a(C_{206}U_{00}+C_{006}U_{20})+\frac{1}{3}\varepsilon {\left(\kappa a\right)}^{2}U_{00}F_{\psi 10}(a)\psi_{\mathrm{eq}10}(a)]{\bar{\sigma }}^{2}\}e_{z},$$

where the coefficients $C_{006}$, $C_{026}$, $C_{116}$, and $C_{206}$ are provided in Equations (A1)–(A4).

### 3.4. Sedimentation Velocity

At the quasi-steady state, the net force acting on the charged soft sphere disappears. Using this constraint after adding Equations (18), (20), and (22), we obtain the sedimentation velocity of the particle inside the charged cavity in the expansion form of Equation (14) with the coefficients as

(23) $$U_{00}=\frac{g}{3\eta aC_{002}}[{r_{0}}^{3}(\rho_{c}-\rho )+(a^{3}-{r_{0}}^{3})(1-\varepsilon_{p})(\rho_{p}-\rho )],$$

(24a) $$U_{01}=0,$$

(24b) $$U_{10}=0,$$

$$U_{02}=-\frac{U_{00}}{C_{006}}[C_{026}+\frac{\varepsilon \kappa^{2}a}{3\eta }F_{\psi 01}(a)\psi_{\mathrm{eq}01}(a)$$

(25a) $$-\frac{2\varepsilon }{3\eta }F_{\psi 01}(a)\frac{d\psi_{\mathrm{eq}01}}{dr}(a)-\frac{\varepsilon a}{3\eta }\frac{dF_{\psi 01}}{dr}(a)\frac{d\psi_{\mathrm{eq}01}}{dr}(a)],$$

$$U_{11}=-\frac{U_{00}}{C_{006}}\{C_{116}+\frac{\varepsilon \kappa^{2}a}{3\eta }[F_{\psi 10}(a)\psi_{\mathrm{eq}01}(a)+F_{\psi 01}(a)\psi_{\mathrm{eq}10}(a)]$$

$$-\frac{2\varepsilon }{3\eta }[F_{\psi 10}(a)\frac{d\psi_{\mathrm{eq}01}}{dr}(a)+F_{\psi 01}(a)\frac{d\psi_{\mathrm{eq}10}}{dr}(a)]$$

(25b) $$-\frac{\varepsilon a}{3\eta }[\frac{dF_{\psi 10}}{dr}(a)\frac{d\psi_{\mathrm{eq}01}}{dr}(a)+\frac{dF_{\psi 01}}{dr}(a)\frac{d\psi_{\mathrm{eq}10}}{dr}(a)]\},$$

$$U_{20}=-\frac{U_{00}}{C_{006}}[C_{206}+\frac{\varepsilon \kappa^{2}a}{3\eta }F_{\psi 10}(a)\psi_{eq10}(a)$$

(25c) $$-\frac{2\varepsilon }{3\eta }F_{\psi 10}(a)\frac{d\psi_{\mathrm{eq}10}}{dr}(a)-\frac{\varepsilon a}{3\eta }\frac{dF_{\psi 10}}{dr}(a)\frac{d\psi_{\mathrm{eq}10}}{dr}(a)],$$

where $U_{00}$ is the settling velocity of an uncharged soft spherical particle inside a concentric uncharged spherical cavity [40], which is positive but smaller than that in an unbounded fluid. As expected, the correction to the sedimentation velocity of the charged soft sphere begins with the second order of the fixed charge densities.

The sedimentation velocity of a charged soft spherical particle within a charged cavity provided by Equations (14) and (23)–(25) can also be expressed as

(26) $$U=U_{00}[1+H_{1}{\left(\kappa a\right)}^{4}{\bar{Q}}^{2}+H_{2}{\left(\kappa a\right)}^{3}\bar{Q}\bar{\sigma }+H_{3}{\left(\kappa a\right)}^{2}{\bar{\sigma }}^{2}],$$

where

(27a) $$H_{1}=\frac{U_{02}}{U_{00}{\left(\kappa a\right)}^{4}},$$

(27b) $$H_{2}=\frac{U_{11}}{U_{00}{\left(\kappa a\right)}^{3}},$$

(27c) $$H_{3}=\frac{U_{20}}{U_{00}{\left(\kappa a\right)}^{2}}.$$

Note that $(\kappa a)\bar{\sigma }$ and ${\left(\kappa a\right)}^{2}\bar{Q}$ do not depend on κ or $n^{\infty }$. For a soft sphere undergoing sedimentation inside a cavity filled with a symmetric electrolyte solution, the dimensionless second-order coefficients $H_{1}$, $H_{2}$, and $H_{3}$ are functions of the ratios of core-to-particle radii $r_{0}/a$, particle-to-cavity radii a/b, particle radius-to-Debye length κa, and particle radius-to-porous-layer-permeation length λa.

The terms involving the coefficients $H_{1}$ and $H_{3}$ can be viewed as the corrections to the sedimentation of a charged soft spherical particle inside a concentric uncharged spherical cavity (σ=0) and an uncharged soft particle (Q=0) inside a concentric charged cavity, respectively. The surface charges of the cavity wall alter the settling velocity of the soft sphere by means of an electroosmotic recirculating flow developed from interactions of the electrical potential gradient caused by the sedimentation with the electrical double layer adjoining the cavity wall and a wall-induced electrical potential on the soft sphere. In the limiting case of $r_{0}/a=0$, Equation (26) reduces to the sedimentation velocity formula for a charged porous spherical particle within a concentric charged cavity [27].

## 4. Results and Discussion

The sedimentation velocity of a charged soft spherical particle within a concentric charged spherical cavity full of the solution of a symmetric electrolyte is expressed by Equations (26) and (27) as a power expansion of the fixed charge densities of its porous surface layer Q and the cavity wall σ up to the second orders $Q^{2}$, Qσ, and $\sigma^{2}$.

### 4.1. The Coefficients $H_{1}$, $H_{2}$, and $H_{3}$

**Figure 2:**
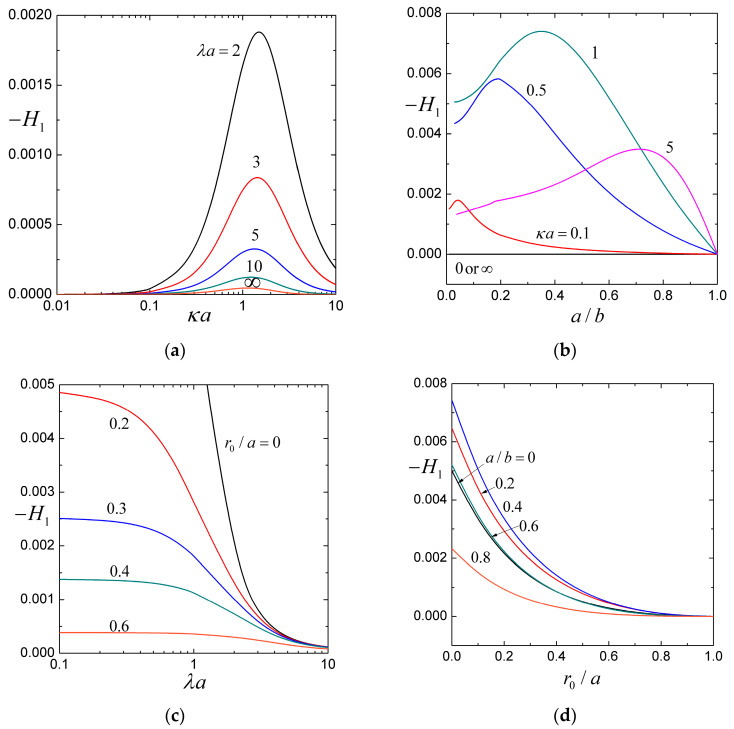
The coefficient $H_{1}$ (accounting for the effect of the particle charge) in Equation (26) for the sedimentation of a soft particle in a cavity full of aqueous KCl solution: (**a**) versus the ratio of particle radius to Debye length κa with $r_{0}/a=0$ and a/b=0.5; (**b**) versus the particle-to-cavity radius ratio a/b with $r_{0}/a=0$ and λa=1; (**c**) versus the ratio of particle radius to porous layer permeation length λa with κa=1 and a/b=0.5; (**d**) versus the core-to-particle radius ratio $r_{0}/a$ with κa=1 and λa=1.

**Figure 3:**
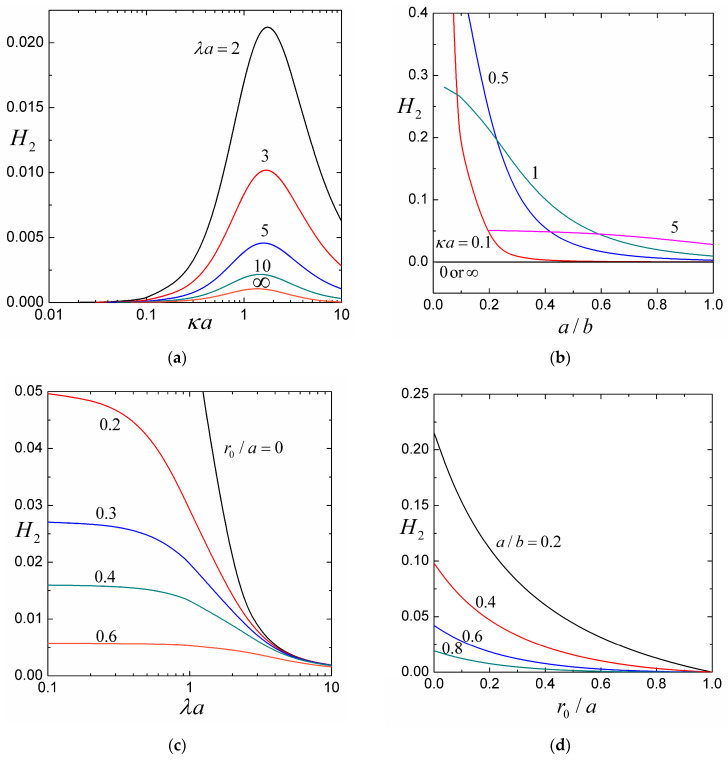
The coefficient $H_{2}$ (accounting for the coupling effect of the fixed charges) in Equation (26) for the sedimentation of a soft particle in a cavity full of aqueous KCl solution: (**a**) versus the ratio of particle radius-to-Debye length κa with $r_{0}/a=0$ and a/b=0.5; (**b**) versus the particle-to-cavity radius ratio a/b with $r_{0}/a=0$ and λa=1; (**c**) versus the ratio of particle radius-to-porous-layer-permeation length λa with κa=1 and a/b=0.5; (**d**) versus the core-to-particle radius ratio $r_{0}/a$ with κa=1 and λa=1.

**Figure 4:**
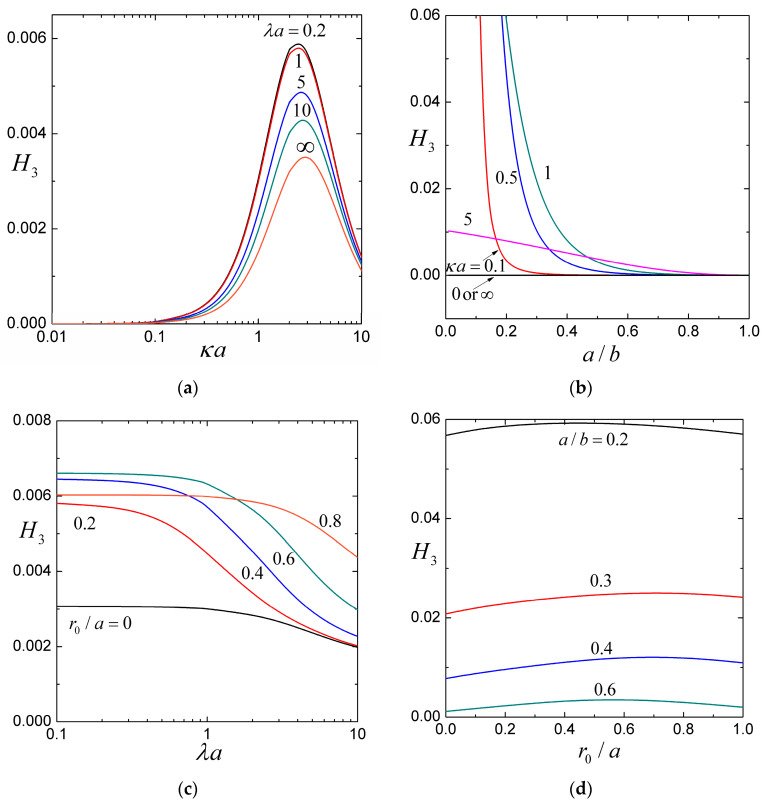
The coefficient $H_{3}$ (accounting for the effect of the cavity charge) in Equation (26) for the sedimentation of a soft particle in a cavity full of aqueous KCl solution: (**a**) versus the ratio of particle radius-to-Debye length κa with $r_{0}/a=0$ and a/b=0.5; (**b**) versus the particle-to-cavity radius ratio a/b with $r_{0}/a=0$ and λa=1; (**c**) versus the ratio of particle radius-to-porous layer permeation length λa with κa=1 and a/b=0.5; (**d**) versus the core-to-particle radius ratio $r_{0}/a$ with κa=1 and λa=1.

For the settling of a soft particle within a cavity filled with an aqueous solution of potassium chloride (KCl, with $\varepsilon k^{2}T^{2}/\eta D_{\pm }Z^{2}e^{2}=0.26$ at room temperature), the coefficients $H_{1}$, $H_{2}$, and $H_{3}$, calculated from Equations (25) and (27), are plotted versus the ratios of particle-to-cavity radii a/b, particle radius-to-Debye length κa, particle radius-to-porous-layer-permeation length λa, and core-to-particle radii $r_{0}/a$ in Figure 2, Figure 3 and Figure 4 for a wide range.

The coefficient $H_{1}$ (and $U_{02}$) is negative, so the presence of stationary space charges in the porous surface layer of the soft sphere reduces its sedimentation velocity. This retarding effect is caused by the electrophoresis in the opposite direction generated by the sedimentation-induced electrical potential field, as provided by Equation (16). For specified values of λa, κa, and $r_{0}/a$, as shown in Figure 2b,d, $-H_{1}$ first increases with an increase in the particle-to-cavity radius ratio a/b, reaches a maximum, and then decreases with a further increase in a/b. It is understood that both $-U_{02}$ and $U_{00}$, provided by Equations (23) and (25a), are monotonic-decreasing functions of a/b, due to the electrophoretic and viscous retardation effects, respectively, of the cavity wall on the moving soft sphere. Compared to $U_{00}$, $-U_{02}$ decreases slow for a low value of a/b and fast for a high value of a/b, causing $-H_{1}$ to reach a maximum according to Equation (27a). As κa increases or λa decreases, the position of this maximum moves to larger a/b.

Both coefficients $H_{2}$ and $H_{3}$ are positive, so the contribution of $H_{3}$ from $\sigma^{2}$ increases the settling velocity of an uncharged soft sphere in the cavity. The counterion concentration in the electric double layer adjoining the cavity wall near the front side of the settling particle increases because of the squeezing of the fluid, while the counterion concentration near the back side of the soft sphere decreases due to fluid compensation, generating a sedimentation-induced electrical potential gradient and electroosmosis along the cavity wall to enhance the recirculating flow and particle sedimentation. The contribution of $H_{2}$ of interactions between the stationary space charge of the porous surface layer of the soft sphere and the surface charge of the cavity wall increases the sedimentation velocity if these charges have the same sign (Qσ>0), while counterions near the particle surface and cavity wall are reduced by mutual competition, thereby dropping the sedimentation-induced electric field and electrophoretic retardation and decreases the particle velocity if these charges are opposite in sign (Qσ<0), and the enrichment of the counterions in the double layers enhances the induced potential gradient and electrophoretic retardation. For the provided values of λa, κa, and $r_{0}/a$, as shown in Figure 3b,d and Figure 4b,d, the coefficients $H_{2}$ and $H_{3}$ are decreasing functions of the particle-to-cavity radius ratio a/b (because the increase of a/b decreases the surface area of the cavity for a particular particle and, therefore, reduces the effect of cavity surface charge on the settling particle).

For the fixed values of λa, a/b, and $r_{0}/a$, as shown in Figure 2a, Figure 3a, and Figure 4a, the coefficients $-H_{1}$, $H_{2}$, and $H_{3}$ have maxima at some finite values of the ratio of particle radius to Debye length κa and gradually fade as κa becomes smaller or larger. In general, as a/b increases, the location of these maxima shifts to larger κa but is insensitive to changes in λa. In the limiting case of very thick double layers (κa→0), the counterions in them are negligible and the particle sedimentation is not affected by the interaction between the space charge of the porous surface layer of the soft sphere and the surface charge of the cavity wall. In the case of very thin double layers (κa→∞), the charge density vanishes everywhere in fluid and the interaction between fixed charges disappears.

For specified values of a/b, $r_{0}/a$, and κa, as shown in Figure 2a,c, Figure 3a,c and Figure 4a,c, the coefficients $-H_{1}$, $H_{2}$, and $H_{3}$ are decreasing functions of the ratio of particle radius-to-porous-layer-permeation length λa as expected. Although $H_{3}$ is only a relatively weak function of λa (the charged cavity wall effect on the settling of the soft particle principally through the electroosmotic recirculating flow has not much to do with the relative permeability of its porous surface layer), $H_{1}$ and $H_{2}$ can strongly depend on λa.

For provided values of κa, λa, and a/b, as shown in Figure 2c,d and Figure 3c,d, the coefficients $-H_{1}$ and $H_{2}$ decrease monotonically and substantially with an increase in the core-to-particle radius ratio $r_{0}/a$ (i.e., a decrease in the relative volume, and thus total space charge, of the porous surface layer of the soft sphere), as expected. On the other hand, as shown in Figure 4c,d, the coefficient $H_{3}$, due to the surface charge of the cavity wall (it has little to do with the space charge of the porous surface layer), can be a relatively weak nonmonotonic function of $r_{0}/a$. It is understood that, as $r_{0}/a$ increases, both $U_{00}$ and $U_{20}$, provided by Equations (23) and (25c), decrease due to the effects of viscous retardation and mainly electroosmotic recirculation, respectively, of the cavity wall on the moving soft sphere. Compared to $U_{00}$, $U_{20}$ decreases slower for low values of $r_{0}/a$ and faster for high values of $r_{0}/a$, causing $H_{3}$ to reach a maximum according to Equation (27c).

### 4.2. The Normalized Sedimentation Velocity

**Figure 5:**
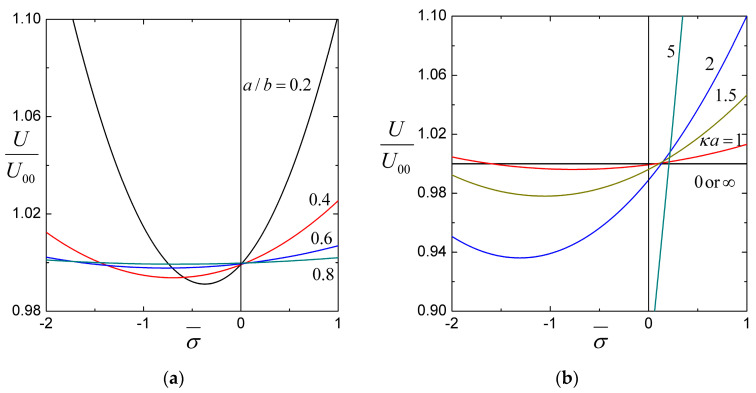

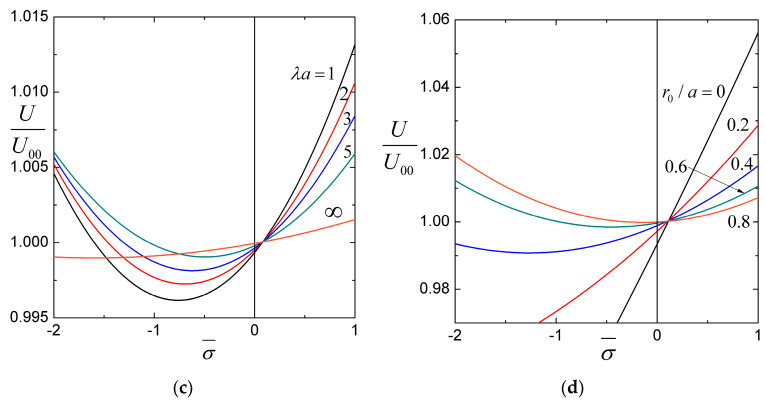
The normalized sedimentation velocity *U*/*U*_00_ of a soft particle with the dimensionless space charge density $\bar{Q}=1$ in a cavity full of aqueous KCl solution versus the dimensionless surface charge density $\bar{\sigma }$: (**a**) for several values of the particle-to-cavity radius ratio a/b with $r_{0}/a=0.5$, λa=1, and κa=1; (**b**) for several values of the ratio of particle radius to Debye length κa with $r_{0}/a=0.5$, a/b=0.5, and λa=1; (**c**) for several values of the ratio of particle radius to porous layer permeation length λa with $r_{0}/a=0.5$, a/b=0.5, and κa=1; (**d**) for several values of the core-to-particle radius ratio $r_{0}/a$ with a/b=0.5, λa=1, and κa=1.

Our results in Figure 2, Figure 3 and Figure 4 show that the coefficient $H_{2}$ in Equation (26) is an order of magnitude larger than the other coefficients $-H_{1}$ and $H_{3}$. Consequently, for a charged soft spherical particle migrating inside a spherical cavity with a surface charge of the same sign (Qσ>0), the net effect of these coefficients is generally an increase of the sedimentation velocity of the particle (with exceptions occurring as $\bar{\sigma }$ is much smaller than $\bar{Q}$ in magnitude). For the case of the cavity with a surface charge of the opposite sign (Qσ<0), if $\left|\bar{\sigma }\right|$ is sufficiently larger/smaller than $\left|\bar{Q}\right|$, the net effect is to enhance/reduce the settling velocity. This trend is manifested in Figure 5, which is a plot of the normalized particle velocity $U/U_{00}$ of a soft spherical particle with a space charge density in its porous surface layer $\bar{Q}=1$ inside a spherical cavity full of an aqueous solution of potassium chloride calculated using Equation (26) versus the surface charge density $\bar{\sigma }$ of the cavity wall. The enhancement and reduction of the particle velocity caused by the cavity wall can be substantial if $\left|\bar{\sigma }\right|$ is relatively large, especially in cases of small core-to-particle radius ratio $r_{0}/a$, ratio of particle radius-to-porous-layer-permeation length λa, and particle-to-cavity radius ratio a/b, with moderate values of the ratio of the particle radius to Debye length κa.

Figure 3b,d, Figure 4b,d and Figure 5a show that the influence of the charged cavity wall on the sedimentation of the soft sphere increases as a/b decreases, with other parameters remaining constant. This result reflects the fact that for a fixed size particle, the reduction of a/b increases the surface area of the cavity and, therefore, enhances the effect of its surface charge on the settling particle. Figure 5b,c,d illustrates that the effect of the cavity wall surface charge on the sedimentation of the soft sphere increases with decreases in $r_{0}/a$ and λa but is not a monotonic function of κa.

## 5. Conclusions

This paper presents an analysis of the sedimentation of a soft spherical particle inside a concentric spherical cavity filled with a symmetric electrolyte solution. By using a regular perturbation method with small fixed charge densities of the porous surface layer of the soft sphere Q and cavity wall σ, a set of linearized electrokinetic equations related to the fluid velocity field, electrical potential profile, and ionic electrochemical potential energy distributions are solved with the relaxation effect of the electric double layers. A closed-form formula for the settling velocity of the soft sphere is provided by Equation (26) as a function of the ratios of particle-to-cavity radii a/b, particle radius-to-Debye length κa, particle radius-to-porous-layer-permeation length λa, and core-to-particle radii $r_{0}/a$. Corrections due to the effect of the fixed charges Q and σ on the sedimentation velocity start from the second orders $Q^{2}$, Qσ, and $\sigma^{2}$. The existence of the surface charge on the cavity wall increases the settling velocity of the charged soft sphere, mainly because of the electroosmotic enhancement of recirculating flow within the cavity induced by the sedimentation potential gradient. When Q and σ have the same sign, the particle velocity is generally enhanced by the presence of the cavity. When these fixed charges have opposite signs and σ is sufficiently large/small relative to Q in magnitude, the particle velocity will be enhanced/reduced by the presence of the charged cavity. The effect of σ on the sedimentation of the soft sphere increases with decreasing a/b, λa, and $r_{0}/a$ but has a maximum at some finite values of κa and disappears as κa approaches zero and infinity. Our results might be used to optimize sedimentation processes, design materials with desired settling properties, or study efficient flotation of low-rank coal with poor hydrophobicity [41,42].

## Data Availability

Data are contained within the article.

## Appendix A

Some functions in Equations (15)–(17) are listed here. In Equation (15),

(A1a) $$F_{00r}(r)=C_{001}+[C_{002}+C_{003}\alpha (\lambda r)+C_{004}\beta (\lambda r)]{\left(\frac{a}{r}\right)}^{3}\;\mathrm{for}\;r_{0}<r<a,$$

(A1b) $$F_{00r}(r)=C_{005}+C_{006}\frac{a}{r}+C_{007}{\left(\frac{a}{r}\right)}^{3}+C_{008}{\left(\frac{r}{a}\right)}^{2}\;\;\;\mathrm{for}\;a<r<b,$$

(A2a) $$F_{p00}(r)=[\frac{1}{2}C_{002}{\left(\frac{a}{r}\right)}^{2}-C_{001}\frac{r}{a}]{\left(\lambda a\right)}^{2}\;\;\;\;\;\;\;\mathrm{for}\;r_{0}<r<a,$$

(A2b) $$F_{p00}(r)=C_{006}{\left(\frac{a}{r}\right)}^{2}+10C_{008}\frac{r}{a}\;\;\;\;\;\;\;\;\;\mathrm{for}\;a<r<b;$$

$$F_{ijr}(r)=C_{ij1}+[C_{ij2}+C_{ij3}\alpha (\lambda r)+C_{ij4}\beta (\lambda r)]{\left(\frac{a}{r}\right)}^{3}+\frac{2}{3{\left(\lambda a\right)}^{2}}\{J_{ij}^{\left(0\right)}(r_{0},r)$$

(A3a) $$-{\left(\frac{a}{r}\right)}^{3}J_{ij}^{\left(3\right)}(r_{0},r)+\frac{3\alpha (\lambda r)}{{\left(\lambda r\right)}^{3}}J_{ij}^{\beta }(r_{0},r)-\frac{3\beta (\lambda r)}{{\left(\lambda r\right)}^{3}}J_{ij}^{\alpha }(r_{0},r)\}\;\mathrm{for}\;r_{0}<r<a,$$

$$F_{ijr}(r)=C_{ij5}+\frac{1}{3}J_{ij}^{\left(2\right)}(a,r)+\frac{a}{r}[C_{ij6}-\frac{1}{3}J_{ij}^{\left(3\right)}(a,r)]$$

(A3b) $$+{\left(\frac{a}{r}\right)}^{3}[C_{ij7}+\frac{1}{15}J_{ij}^{\left(5\right)}(a,r)]+{\left(\frac{r}{a}\right)}^{2}[C_{ij8}-\frac{1}{15}J_{ij}^{\left(0\right)}(a,r)]\;\mathrm{for}\;a<r<b,$$

(A4a) $$F_{pij}(r)={\left(\lambda a\right)}^{2}[\frac{1}{2}C_{ij2}{\left(\frac{a}{r}\right)}^{2}-C_{ij1}\frac{r}{a}]-\frac{1}{3}{\left(\frac{a}{r}\right)}^{2}J_{ij}^{\left(3\right)}(r_{0},r)-\frac{2r}{3a}J_{ij}^{\left(0\right)}(r_{0},r)\;\mathrm{for}\;r_{0}<r<a,$$

(A4b) $$F_{pij}(r)={\left(\frac{a}{r}\right)}^{2}[C_{ij6}-\frac{1}{3}J_{ij}^{\left(3\right)}(a,r)]+\frac{r}{a}[10C_{ij8}-\frac{2}{3}J_{ij}^{\left(0\right)}(a,r)]\;\mathrm{for}\;a<r<b,$$

where (i, j) equal (0, 2), (1, 1), and (2, 0),

(A5) $$J_{ij}^{\left(n\right)}(r_{1},r_{2})=\int_{r_{1}}^{r_{2}}{\left(\frac{r}{a}\right)}^{n}G_{ij}(r)dr,$$

(A6a) $$J_{ij}^{\alpha }(r_{1},r_{2})=\int_{r_{1}}^{r_{2}}\alpha (\lambda r)G_{ij}(r)dr,$$

(A6b) $$J_{ij}^{\beta }(r_{1},r_{2})=\int_{r_{1}}^{r_{2}}\beta (\lambda r)G_{ij}(r)dr,$$

(A7a) $$G_{02}(r)=\frac{\varepsilon {\left(\kappa a\right)}^{2}}{2\eta r}[F_{01+}(r)-F_{01-}(r)]\frac{d\psi_{\mathrm{eq}01}}{dr},$$

(A7b) $$G_{11}(r)=\frac{\varepsilon {\left(\kappa a\right)}^{2}}{2\eta r}\{[F_{10+}(r)-F_{10-}(r)]\frac{d\psi_{\mathrm{eq}01}}{dr}+[F_{01+}(r)-F_{01-}(r)]\frac{d\psi_{\mathrm{eq}10}}{dr}\},$$

(A7c) $$G_{20}(r)=\frac{\varepsilon {\left(\kappa a\right)}^{2}}{2\eta r}[F_{10+}(r)-F_{10-}(r)]\frac{d\psi_{\mathrm{eq}10}}{dr},$$

(A8a) α(x)=xcoshx−sinhx,

(A8b) β(x)=xsinhx−coshx,

and expressions of the coefficients $C_{00n}$, $C_{02n}$, $C_{11n}$, and $C_{20n}$ with n=1, 2, …, and 8 are lengthy and provided in [43].

In Equations (16) and (17),

$$F_{ij\pm }(r)=\pm \frac{1}{3D_{\pm }(b^{3}-r_{0}^{3})}\{a^{3}r[2+{\left(\frac{r_{0}}{r}\right)}^{3}][G_{3}(a,b)+G_{3}(r_{0},a)]+b^{3}r[1+\frac{1}{2}{\left(\frac{r_{0}}{r}\right)}^{3}][G_{0}(a,b)$$

(A9a) $$+G_{0}(r_{0},a)]+(b^{3}-r_{0}^{3})[a^{3}r^{-2}G_{3}(r_{0},r)-rG_{0}(r_{0},r)]\}\;\mathrm{for}\;r_{0}<r<a,$$

$$F_{ij\pm }(r)=\pm \frac{1}{3D_{\pm }(b^{3}-r_{0}^{3})}[a^{3}r\{[2+{\left(\frac{b}{r}\right)}^{3}]G_{3}(r_{0},a)+[2+{\left(\frac{r_{0}}{r}\right)}^{3}]G_{3}(a,b)\}+r\{r_{0}^{3}[1+\frac{1}{2}{\left(\frac{b}{r}\right)}^{3}]G_{0}(r_{0},a)$$

(A9b) $$+b^{3}[1+\frac{1}{2}{\left(\frac{r_{0}}{r}\right)}^{3}]G_{0}(a,b)\}+(b^{3}-r_{0}^{3})\{a^{3}r^{-2}G_{3}(a,r)-rG_{0}(a,r)\}]\;\mathrm{for}\;a<r<b,$$

$$F_{\psi ij}(r)=\frac{1}{2\kappa r^{2}}[\alpha (\kappa r)B_{\psi ij}(r_{0},r)-\beta (\kappa r)A_{\psi ij}(r_{0},r)]$$

$$+\frac{1}{2W\kappa r^{2}}\{\Omega_{4}\beta (\kappa r)[e^{\kappa r_{0}}\Omega_{1}A_{\psi ij}(r_{0},b)+e^{\kappa (b+r_{0})}\Omega_{2}\{A_{\psi ij}(r_{0},b)-B_{\psi ij}(r_{0},b)\}]$$

(A10a) $$-[\alpha (\kappa r)\Omega_{1}A_{\psi ij}(r_{0},b)-e^{\kappa b}\Omega_{2}\Omega_{5}\{A_{\psi ij}(r_{0},b)-B_{\psi ij}(r_{0},b)\}]\}\;\mathrm{for}\;r_{0}<r<a,$$

$$F_{\psi ij}(r)=\frac{1}{2\kappa r^{2}}[\alpha (\kappa r)B_{\psi ij}(a,r)-\beta (\kappa r)A_{\psi ij}(a,r)]$$

$$-\frac{1}{2W\kappa r^{2}}\{\Omega_{4}\beta (\kappa r)[e^{\kappa r_{0}}\Omega_{1}A_{\psi ij}(a,b)-e^{\kappa (b+r_{0})}\Omega_{2}\{A_{\psi ij}(a,b)-B_{\psi ij}(r_{0},r)\}$$

$$+e^{\kappa b}\Omega_{2}\Omega_{5}A_{\psi ij}(r_{0},a)]+\alpha (\kappa r)[e^{\kappa b}\Omega_{2}\Omega_{3}B_{\psi ij}(a,b)+e^{\kappa r_{0}}\Omega_{1}\Omega_{4}B_{\psi ij}(r_{0},a)$$

(A10b) $$-e^{\kappa (b+r_{0})}\Omega_{2}\Omega_{4}B_{\psi ij}(r_{0},b)+\Omega_{5}(\Omega_{1}-e^{\kappa b}\Omega_{2})A_{\psi ij}(r_{0},b)]\}\;\mathrm{for}\;a<r<b,$$

where (i, j) equal (0, 1) and (1, 0),

(A11) $$G_{n}(r_{1},r_{2})=\int_{r_{1}}^{r_{2}}(\frac{r}{a}{)}^{n}\frac{d\psi_{\mathrm{eq}ij}}{dr}F_{00r}(r)dr,$$

(A12a) $$A_{\psi ij}(r_{1},r_{2})=\int_{r_{1}}^{r_{2}}\alpha (\kappa r)[F_{ij+}(r)-F_{ij-}(r)]dr,$$

(A12b) $$B_{\psi ij}(r_{1},r_{2})=\int_{r_{1}}^{r_{2}}\beta (\kappa r)[F_{ij+}(r)-F_{ij-}(r)]dr,$$

(A13) $$W=e^{\kappa b}\Omega_{2}\Omega_{3}-e^{\kappa r_{0}}\Omega_{1}\Omega_{4},$$

(A14a) $$\Omega_{1}=2+\kappa b(2+\kappa b),$$

(A14b) $$\Omega_{2}=2\alpha (\kappa b)-{\left(\kappa b\right)}^{2}\sinh(\kappa b),$$

(A14c) $$\Omega_{3}=2+\kappa r_{0}(2+\kappa r_{0}),$$

(A14d) $$\Omega_{4}=2\alpha (\kappa r_{0})-{\left(\kappa r_{0}\right)}^{2}\sinh(\kappa r_{0}),$$

(A14e) $$\Omega_{5}=\Omega_{3}-e^{\kappa r_{0}}\Omega_{4}$$

## References

[1] Adachi Y. (2016). Sedimentation and Electrophoresis of a Porous Floc and a Colloidal Particle Coated with Polyelectrolytes. Curr. Opin. Colloid Interface Sci..

[2] Satoh A. (2015). Sedimentation Behavior of Dispersions Composed of Large and Small Charged Colloidal Particles: Development of New Technology to Improve the Visibility of Small Lakes and Ponds. Environ. Eng. Sci..

[3] Khair A.S. (2018). Strong Deformation of the Thick Electric Double Layer around a Charged Particle during Sedimentation or Electrophoresis. Langmuir.

[4] Gopmandal P.P., Bhattacharyya S., Barman B. (2014). Effect of Induced Electric Field on Migration of a Charged Porous Particle. Eur. Phys. J. E.

[5] Booth F. (1954). Sedimentation Potential and Velocity of Solid Spherical Particles. J. Chem. Phys..

[6] Stigter D. (1980). Sedimentation of Highly Charged Colloidal Spheres. J. Phys. Chem..

[7] Ohshima H., Healy T.W., White L.R., O’Brien R.W. (1984). Sedimentation Velocity and Potential in a Dilute Suspension of Charged Spherical Colloidal Particles. J. Chem. Soc. Faraday Trans. 2.

[8] Zholkovskiy E.K., Masliyah J.H., Shilov V.N., Bhattacharjee S. (2007). Electrokinetic Phenomena in Concentrated Disperse Systems: General Problem Formulation and Spherical Cell Approach. Adv. Colloid Interface Sci..

[9] Carrique F., Arroyo F.J., Delgado A.V. (2001). Sedimentation Velocity and Potential in a Concentrated Colloidal Suspension: Effect of a Dynamic Stern Layer. Colloids Surf. A.

[10] Keh H.J., Ding J.M. (2000). Sedimentation Velocity and Potential in Concentrated Suspensions of Charged Spheres with Arbitrary Double-Layer Thickness. J. Colloid Interface Sci..

[11] Ohshima H. (1998). Sedimentation Potential in a Concentrated Suspension of Spherical Colloidal Particles. J. Colloid Interface Sci..

[12] Levine S., Neale G., Epstein N. (1976). The Prediction of Electrokinetic Phenomena within Multiparticle Systems II. Sedimentation Potential. J. Colloid Interface Sci..

[13] Keh H.J., Chen W.C. (2006). Sedimentation Velocity and Potential in Concentrated Suspensions of Charged Porous Spheres. J. Colloid Interface Sci..

[14] Hermans J.J. (1955). Sedimentation and Electrophoresis of Porous Spheres. J. Polym. Sci..

[15] Chiu Y.S., Keh H.J. (2014). Sedimentation Velocity and Potential in a Concentrated Suspension of Charged Soft Spheres. Colloids Surf. A.

[16] Ohshima H. (2000). Sedimentation Potential and Velocity in a Concentrated Suspension of Soft Particles. J. Colloid Interface Sci..

[17] Jiemvarangkula P., Zhang W., Lien H.-L. (2011). Enhanced Transport of Polyelectrolyte Stabilized Nanoscale Zero-Valent Iron (nZVI) in Porous Media. Chem. Eng. J..

[18] Lee S.Y., Yalcin S.E., Joo S.W., Sharma A., Baysal O., Qian S. (2010). The Effect of Axial Concentration Gradient on Electrophoretic Motion of a Charged Spherical Particle in a Nanopore. Microgravity Sci. Technol..

[19] Prakash J. (2020). Hydrodynamic mobility of a porous spherical particle with variable permeability in a spherical cavity. Microsyst. Technol..

[20] Lee T.C., Keh H.J. (2013). Slow Motion of a Spherical Particle in a Spherical Cavity with Slip Surfaces. Int. J. Eng. Sci..

[21] Chen S.B., Ye X. (2000). Boundary effect on slow motion of a composite sphere perpendicular to two parallel impermeable plates. Chem. Eng. Sci..

[22] Kim S., Karrila S.J. (2005). Microhydrodynamics: Principles and Selected Applications.

[23] Happel J., Brenner H. (1983). Low Reynolds Number Hydrodynamics.

[24] Pujar N.S., Zydney A.L. (1996). Boundary Effects on the Sedimentation and Hindered Diffusion of Charged Particles. AIChE J..

[25] Lee E., Yen C.-B., Hsu J.-P. (2000). Sedimentation of a Nonconducting Sphere in a Spherical Cavity. J. Phys. Chem. B.

[26] Keh H.J., Cheng T.F. (2011). Sedimentation of a Charged Colloidal Sphere in a Charged Cavity. J. Chem. Phys..

[27] Chang Y.J., Keh H.J. (2013). Sedimentation of a Charged Porous Particle in a Charged Cavity. J. Phys. Chem. B.

[28] Masliyah J.H., Polikar M. (1980). Terminal Velocity of Porous Spheres. Can. J. Chem. Eng..

[29] Matsumoto K., Suganuma A. (1977). Settling Velocity of a Permeable Model Floc. Chem. Eng. Sci..

[30] Aoyanagi O., Muramatsu N., Ohshima H., Kondo T. (1994). Electrophoretic Behavior of PolyA-Graft-PolyB-Type Microcapsules. J. Colloid Interface Sci..

[31] Morita K., Muramatsu N., Ohshima H., Kondo T. (1991). Electrophoretic Behavior of Rat Lymphocyte Subpopulations. J. Colloid Interface Sci..

[32] Kawahata S., Ohshima H., Muramatsu N., Kondo T. (1990). Charge Distribution in the Surface Region of Human Erythrocytes as Estimated from Electrophoretic Mobility Data. J. Colloid Interface Sci..

[33] Ahualli S., Jimenez M.L., Carrique F., Delgado A.V. (2009). AC Electrokinetics of Concentrated Suspensions of Soft Particles. Langmuir.

[34] Lopez-Garcia J.J., Grosse C., Horno J. (2006). Numerical Calculation of the Electrophoretic Mobility of Concentrated Suspensions of Soft Particles. J. Colloid Interface Sci..

[35] Koplik J., Levine H., Zee A. (1983). Velocity Renormalization in the Brinkman Equation. Phys. Fluids.

[36] Neale G., Epstein N., Nader W. (1973). Creeping Flow Relative to Permeable Spheres. Chem. Eng. Sci..

[37] Chen W.J., Keh H.J. (2013). Electrophoresis of a charged soft particle in a charged cavity with arbitrary double-layer thickness. J. Phys. Chem. B.

[38] Makino K., Yamamoto S., Fujimoto K., Kawaguchi H., Ohshima H. (1994). Surface Structure of Latex Particles Covered with Temperature-Sensitive Hydrogel Layers. J. Colloid Interface Sci..

[39] Blaakmeer J., Bohmer M.R., Cohen Stuart M.A., Fleer G.J. (1990). Adsorption of Weak Polyelectrolytes on Highly Charged Surfaces. Poly(Acrylic Acid) on Polystyrene Latex with Strong Cationic Groups. Macromolecules.

[40] Keh H.J., Chou J. (2004). Creeping Motions of a Composite Sphere in a Concentric Spherical Cavity. Chem. Eng. Sci..

[41] Li L., Chen S., Wang S., Tao X., Zhu X., Cheng G., Gui D. (2019). Influence of pickling on the surface composition and flotability of Daliuta long-flame coal. Powder Technol..

[42] Xu F., Wang S., Kong R., Wang C. (2023). Synergistic effects of dodecane-castor oil acid mixture on the flotation responses of low-rank coal: A combined simulation and experimental study. Int. J. Min. Sci. Technol..

[43] Lin Y.J. (2024). Sedimentation of a Charged soft Particle in a Charged Cavity. Master’s Thesis.

